# Supplementary material for: The interactome of intact mitochondria by cross-linking mass spectrometry provides evidence for coexisting respiratory supercomplexes
Source: Mol Cell Proteomics. 2017 Dec 8;17(2):216–32. doi: 10.1074/mcp.RA117.000470 (PMC5795388; doi:10.1074/mcp.RA117.000470)
Supplement: Supplemental Data [file supp_RA117.000470_133922_0_supp_23978_fzffwf.zip › spectra_annotation/mito_DR_spectra_annotation/43-2-13-1-4-1.pdf]

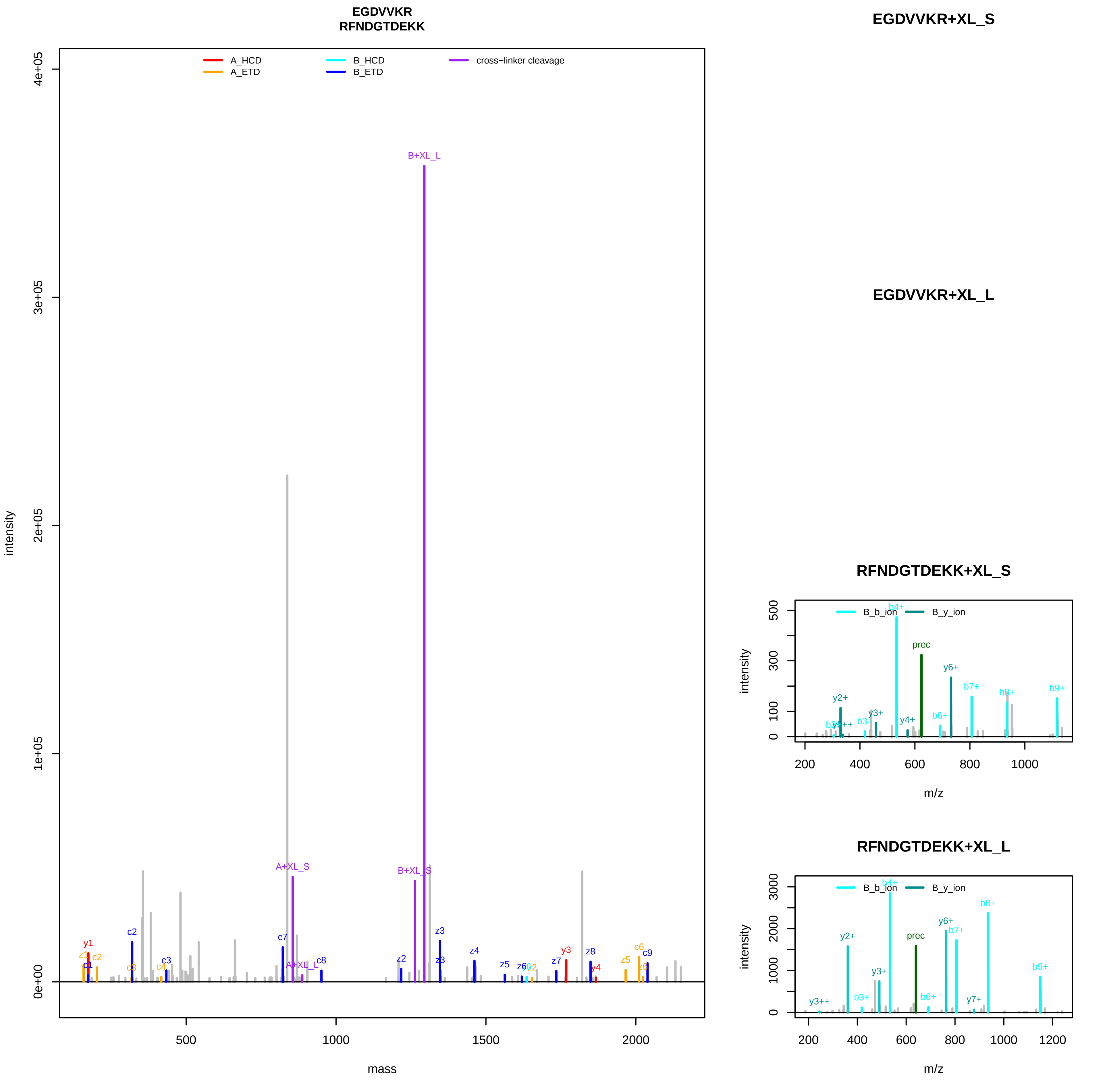

EGDVVKR
RFNDGTDEKK
A_HCD
A_ETD
B_HCD
B_ETD
cross−linker cleavage
intensity
mass
B+XL_L
A+XL_S
B+XL_S
A+XL_L
y1
z1
c1
c2
c3
c4
c7
c8
z2
z3
c3
z4
z5
z6
z7
y3
z8
y4
z5
c6
c9
0e+00
1e+05
2e+05
3e+05
4e+05
500
1000
1500
2000
EGDVVKR+XL_S
EGDVVKR+XL_L
RFNDGTDEKK+XL_S
B_b_ion
B_y_ion
b4+
prec
y6+
b7+
b8+
b9+
y2+
y3+
y4+
b6+
b3+
m/z
RFNDGTDEKK+XL_L
y3++
y7+
0
100
300
500
1000
2000
3000
200
400
600
800
1200
